# A cross-disorder PRS-pheWAS of 5 major psychiatric disorders in UK Biobank

**DOI:** 10.1371/journal.pgen.1008185

**Published:** 2020-05-11

**Authors:** Beate Leppert, Louise A. C. Millard, Lucy Riglin, George Davey Smith, Anita Thapar, Kate Tilling, Esther Walton, Evie Stergiakouli

**Affiliations:** 1 MRC Integrative Epidemiology Unit (IEU), University of Bristol, Bristol, United Kingdom; 2 Population Health Sciences, Bristol Medical School, University of Bristol, Bristol, United Kingdom; 3 Intelligent Systems Laboratory, University of Bristol, Bristol, United Kingdom; 4 Division of Psychological Medicine and Clinical Neurosciences; MRC Centre for Neuropsychiatric Genetics and Genomics, Cardiff University, Cardiff, United Kingdom; 5 Department of Psychology, University of Bath, Bath, United Kingdom; Case Western Reserve University, UNITED STATES

## Abstract

Psychiatric disorders are highly heritable and associated with a wide variety of social adversity and physical health problems. Using genetic liability (rather than phenotypic measures of disease) as a proxy for psychiatric disease risk can be a useful alternative for research questions that would traditionally require large cohort studies with long-term follow up. Here we conducted a hypothesis-free phenome-wide association study in about 330,000 participants from the UK Biobank to examine associations of polygenic risk scores (PRS) for five psychiatric disorders (major depression (MDD), bipolar disorder (BP), schizophrenia (SCZ), attention-deficit/ hyperactivity disorder (ADHD) and autism spectrum disorder (ASD)) with 23,004 outcomes in UK Biobank, using the open-source PHESANT software package. There was evidence after multiple testing (p<2.55x10^-06^) for associations of PRSs with 294 outcomes, most of them attributed to associations of PRS_MDD_ (n = 167) and PRS_SCZ_ (n = 157) with mental health factors. Among others, we found strong evidence of association of higher PRS_ADHD_ with 1.1 months younger age at first sexual intercourse [95% confidence interval [CI]: -1.25,-0.92] and a history of physical maltreatment; PRS_ASD_ with 0.01% lower erythrocyte distribution width [95%CI: -0.013,-0.007]; PRS_SCZ_ with 0.95 lower odds of playing computer games [95%CI:0.95,0.96]; PRS_MDD_ with a 0.12 points higher neuroticism score [95%CI:0.111,0.135] and PRS_BP_ with 1.03 higher odds of having a university degree [95%CI:1.02,1.03]. We were able to show that genetic liabilities for five major psychiatric disorders associate with long-term aspects of adult life, including socio-demographic factors, mental and physical health. This is evident even in individuals from the general population who do not necessarily present with a psychiatric disorder diagnosis.

## Author summary

Psychiatric disorders are associated with a wide range of adverse health, social and economic problems. Our study investigated the association of genetic risk for five common psychiatric disorders with socio- demographics, lifestyle and health of about 330,000 participants in the UK Biobank using a systematic, hypothesis-free approach. We found that genetic risk for attention deficit/hyperactivity disorder (ADHD) and bipolar disorder were most strongly associated with lifestyle factors, such as time of first sexual intercourse and educational attainment. Genetic risks for autism spectrum disorder and schizophrenia were associated with altered blood cell counts and decreased risk of playing computer games, respectively. Increased genetic risk for depression was associated with other mental health outcomes such as neuroticism and irritability. In general, our results suggest that genetic risk for psychiatric disorders associates with a range of health and lifestyle traits that were measured in adulthood, in individuals from the general population who do not necessarily present with a psychiatric disorder diagnosis. However, it is important to note that these associations are not necessary causal but can also represent genetic correlation or be influenced by other factors, such as socio-economic factors and selection into the cohort. The findings should inform future research using causally informative designs.

## Introduction

Family and twin research as well as large-scale genome-wide association studies (GWAS) have shown that psychiatric disorders are highly heritable [1] and that genetic risks for psychiatric disorders are associated with socio-economic factors, physical health outcomes as well as other psychiatric disorders [2–5]. Using genetic liability (rather than phenotypic measures of disease) as a proxy for psychiatric disease risk can be a useful alternative for research questions that would traditionally require long-term follow up and big datasets due to the low prevalence of some of the psychiatric disorders of interest in the population (e.g. adult-onset health consequences of child neurodevelopmental disorders). In addition, while high genetic risk for a psychiatric disorder is not always indicative of a diagnosis of psychiatric disease, it can index underlying subthreshold symptomatology that can still impact later adversities and quality of life [6]. Furthermore, psychiatric diagnosis may not always be available in any cohort, e.g. there are only very few self-reported diagnosis of Attention deficit/hyperactivity disorder (ADHD), autism spectrum disorder (ASD) or schizophrenia (SCZ) available in the UK Biobank sample, which would make direct comparisons between participants with and without a diagnosis impossible.

So far, studies have used hypothesis-driven approaches to investigate associations of genetic risk for psychiatric disorders with various psychiatric and health outcomes as well as lifestyle factors [7,8]. However, big data resources that are readily available, such as UK Biobank with about 500,000 participants, provide rich phenotypic information that can be used for hypothesis-free studies and offset the multiple testing burden. Phenome-wide association studies (pheWAS) are a type of hypothesis-free analysis where the association of a trait of interest is systematically tested with a potentially large number of phenotypes and can be hypothesis-generating by identifying an association when there is no prior reason to expect that an association may exist. As all available phenotypes are tested and the less ‘significant’ results published alongside those of greater ‘significance’, pheWAS can help to reduce biases associated with hypothesis-driven studies where researchers might only publish the most desirable or expected results.

In a Polygenic Risk Score (PRS) pheWAS (PRS-pheWAS) genetic risk is used as a proxy for lifelong liability for a disorder to explore associations of this genetic liability with a broad range of traits. Understanding these associations will be essential to inform prevention or early intervention strategies. However, conclusions about causality are limited due to the low predictive power and high pleiotropic effects of genetic risk scores for psychiatric conditions [8].

The aim of this study was to investigate the associations between genetic risk for five common psychiatric disorders–attention-deficit/ hyperactivity disorder (ADHD), autism spectrum disorder (ASD), schizophrenia (SCZ), major depression (MDD) and bipolar disorder (BP)—with a wide range of socio-demographic, lifestyle, physical and mental health outcomes in UK Biobank, using the systematic hypothesis-free PRS-pheWAS approach.

## Results

**Fig 1:**
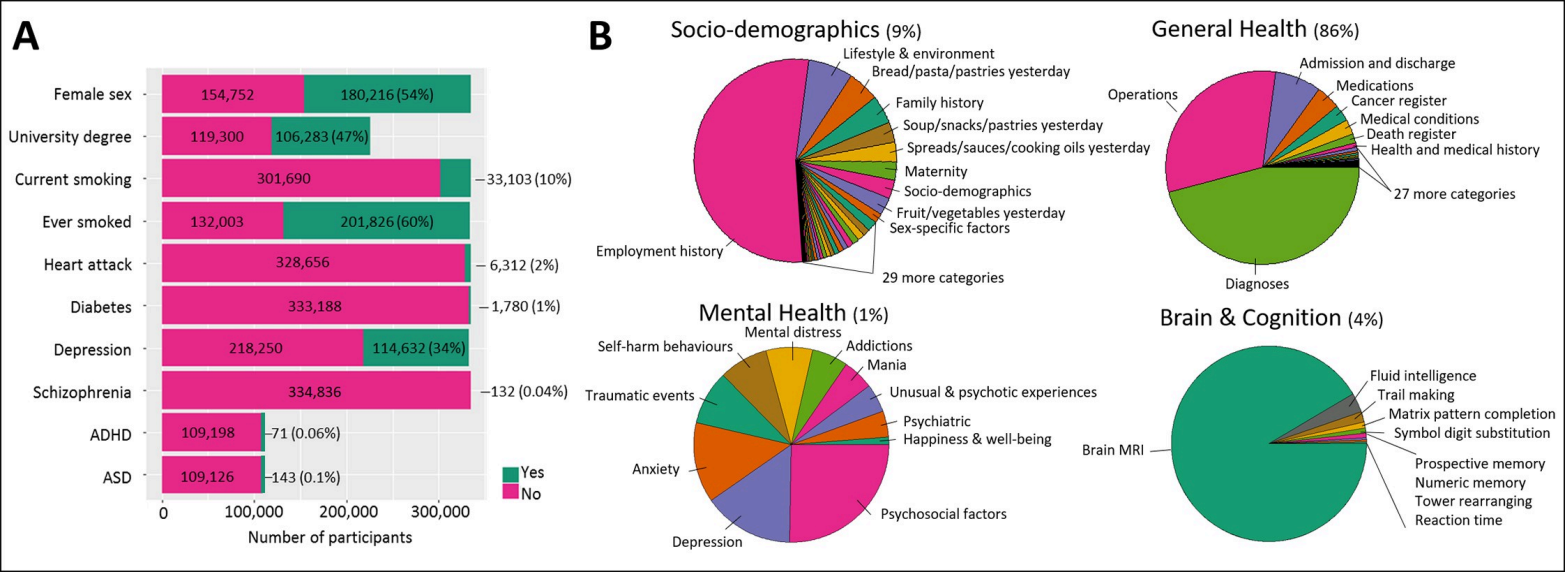
Study overview. (A) Descriptive overview of selected outcomes in UK Biobank. (B) Categories of UK Biobank with the size of pie chart sections indicating the number of included outcomes: socio-demographics (n = 2,057), general health (n = 19,740), mental health (n = 233), brain and cognition (n = 974).

In total 334,976 participants of white British ancestry in UK Biobank were included in this study with an average age of 56 (standard deviation [SD] = 8) years. A descriptive overview of selected UK Biobank study sample characteristics is given in Fig 1A. The UK Biobank participants are known to be more educated and healthier than the average UK population which is reflected in the high percentage of people with a university degree (47%) and low prevalence of current smoking (10%) in the sample, which is comparable to the full UK Biobank release [9]. Furthermore, 34% of participants reported to have seen a general practitioner and 11% a psychiatrist for nerves, anxiety, tension or depression but there are few self-reported cases of schizophrenia (n = 132), ADHD (n = 71), ASD (n = 143) or bipolar disorder (n = 439). An overview of UK Biobank phenotype categories is given in Fig 1B.

The low number of self-reported ADHD, ASD, schizophrenia and bipolar disorder cases did not allow a direct test of predictive power for the respective PRS and we relied on the predictive accuracy reported in other studies [10–14]. The broad question whether participants have “Seen a psychiatrist for nerves, anxiety, tension or depression” was predicted by the PRS_MDD_ (OR: 1.09 [95% confidence interval [CI]: 1.08,1.10] p = 5x10^-52^) and PRS_SCZ_ (OR: 1.05 [95% CI:1.04,1.06] p = 2x10^-17^).

### Disorder specific effects

**Fig 2:**
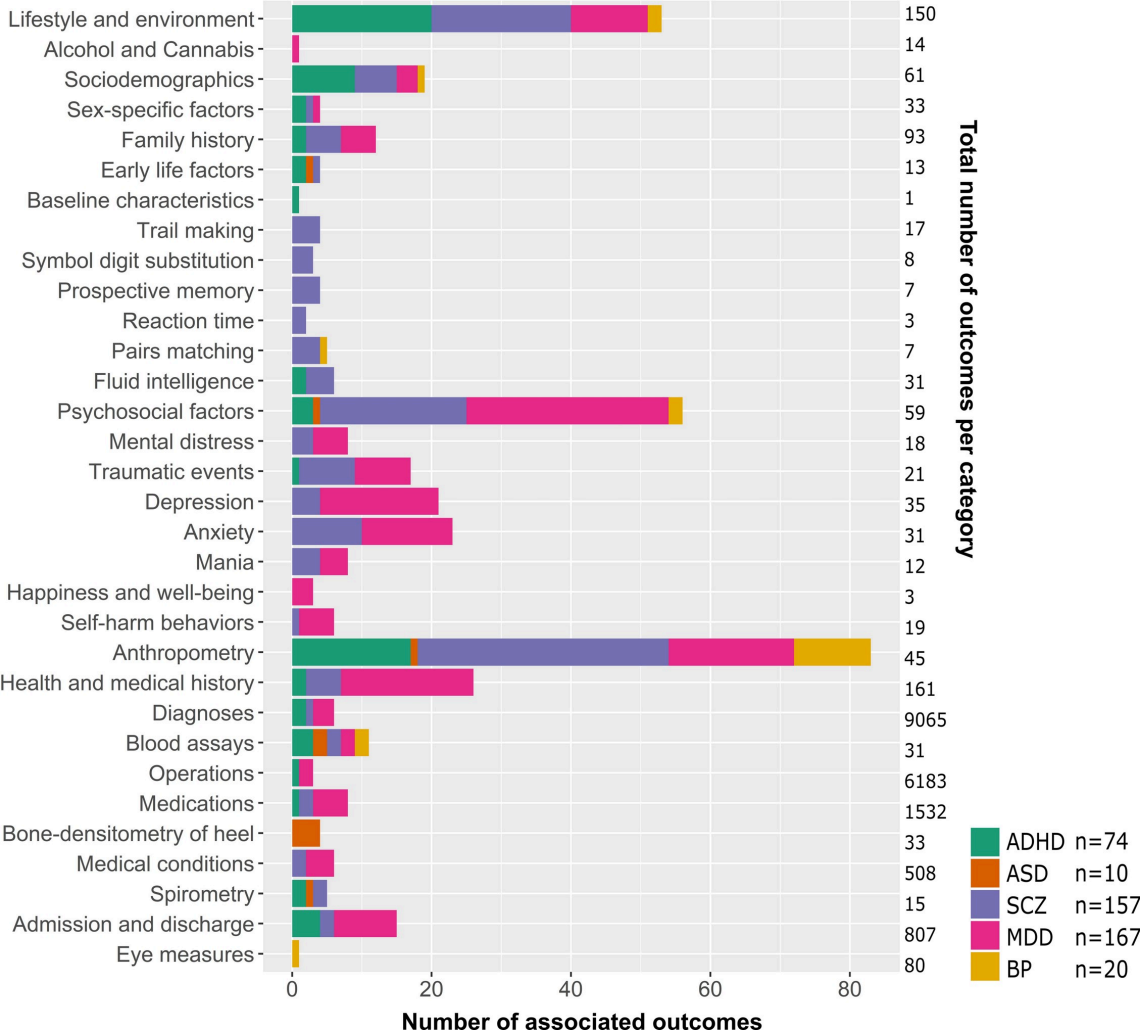
Overview of the distribution of disorder specific polygenic risk score (p<5x10^-8^) associated outcomes per category of the UK Biobank variables catalogue. Shown are the number of associations with polygenic risk scores for attention deficit/hyperactivity disorder (ADHD), autism spectrum disorder (ASD), schizophrenia (SCZ), major depression (MDD) and bipolar disorder (BP).

The PRS-pheWAS of each psychiatric disorder tested the association of the respective polygenic risk score, aggregated from independent, genome-wide significant SNPs, with 23,004 outcomes in UK Biobank, adjusted for age, sex and the first 10 genetic principal components. There was strong evidence after multiple testing correction based on the number of independent tests derived from spectral decomposition (p<2.55x10^-6^) for associations of either the ADHD, ASD, SCZ, MDD or BP PRS with 294 outcomes in 37 UK Biobank categories (Fig 2 and S1 Table) as described below. Of those, 290 outcomes also pass the more stringent Bonferroni threshold (2.17x10^-6^). Correlations among the PRS can be found in supplementary S2 Table. A detailed list of all PRS-pheWAS results generated by the open-source PHESANT software package can be found in S3 Table. Unless stated as a PHESANT result, estimates for continuous outcomes were generated by follow-up linear regressions to compute estimates on their original scale, as PHESANT automatically applied an inverse normal rank conversion to all continuous outcomes.

#### Attention deficit/ hyperactivity disorder

**Fig 3:**
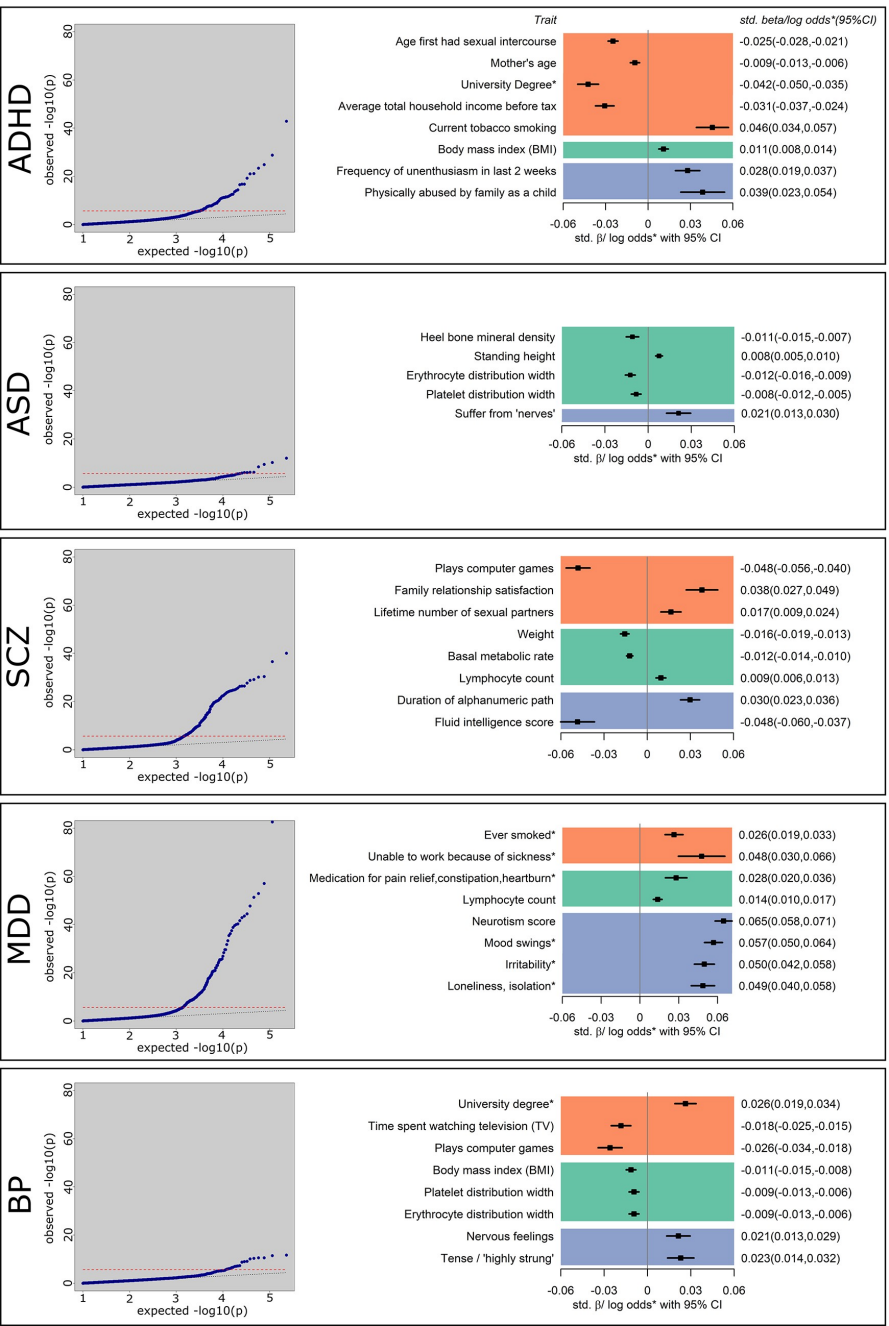
PRS-PheWAS results for attention deficit/hyperactivity disorder (ADHD), autism spectrum disorder (ASD), schizophrenia (SCZ), major depressive disorder (MDD) and bipolar disorder (BP). Left hand panel: QQ plots of expected versus observed p-values for association of PRS with all outcomes in UK Biobank. Red line indicates the significance threshold derived by spectral decomposition (2.5x10^-6^). Right hand panel: selected results from different categories with p-values below the significance threshold and estimates generated by PHESANT. Results for continuous outcomes (std. β) are the standard deviation change of inverse-rank normal transformed outcome per 1 SD higher PRS.

PRS_ADHD_ was strongly associated with 74 outcomes (Fig 3) including 36 socio-demographic factors, 32 general health and 6 mental health, brain and cognition outcomes. The strongest evidence of association with PRS_ADHD_ was seen for socio-demographic and lifestyle factors. 1 SD higher PRS_ADHD_ was associated with a 1.09 month younger age at first sexual intercourse [95% CI: -1.25,-0.92] (p = 2.0x10^-16^), and 0.96 lower odds of having a university degree [95% CI: 0.95, 0.97] (p = 1.7x10^-29^). In addition, higher PRS_ADHD_ was associated with younger age of their parents (-0.08 years [95%CI: -0.102,-0.050] p = 5.1x10^-9^; -0.10 years [95% CI: -0.136,-0.069] p = 1.9x10^-9^, for mother and father respectively), 0.97 lower odds of average household income [95%CI: 0.96,0.98] (p = 5.7x10^-20^), 1.05 higher odds of current smoking [95%CI: 1.03,1.06] (p = 5.7x10^-15^) and 1.04 higher odds of experiencing physical abuse as a child [95%CI: 1.02,1.06] (p = 4.4x10^-6^).

Further, 1 SD increase in PRS_ADHD_ was associated with 15 physical health outcomes related to obesity, including 0.05 kg/m^2^ higher BMI [95%CI: 0.036,0.089] (p = 1.7x10^-10^), leg and arm fat mass, waist circumference and trunk fat mass. Furthermore, there was evidence for an association of PRS_ADHD_ with blood measures, such as 0.02 cells/L higher leukocyte count [95%CI: 0.011,0.025] (p = 2.5x10^-7^).

Associations seen for brain and cognition include 0.04 points lower fluid intelligence score [95%CI: -0.051,-0.026] (p = 1.9x10^-9^).

#### Autism spectrum disorder

PRS_ASD_ was strongly associated with 10 outcomes (Fig 3), including 1 socio-demographic, 8 general health and 1 mental health outcome.

The strongest association of PRS_ASD_ was found for lower erythrocyte distribution width where 1 SD higher PRS_ASD_ associated with 0.01% lower erythrocyte distribution width [95% CI: -0.013, -0.007] (p = 6.3x10^-10^) and 0.98 lower odds of comparative body size at age 10 [95%CI:0.97,0.98] (p = 6.6x10^-11^). Furthermore, 1 SD higher PRS_ASD_ was associated with 0.001 g/cm^2^ lower heel bone mineral density (BMD) [95%CI:-0.002,-0.001] (p = 4.0x10^-5^).

The only mental health outcome that was associated with PRS_ASD_ was 1.02 higher odds of being a nervous person (“suffer from nerves”) [95%CI:1.01,1.03] (p = 7.9x10^-7^).

#### Schizophrenia

There was strong evidence of association for PRS_SCZ_ with 157 outcomes (Fig 3), including 33 socio-demographic, 72 mental health and cognition and 52 general health outcomes.

The strongest evidence of an association with higher PRS_SCZ_ was detected for time of completing an online cognitive function test (pairs matching) (231msec [95%CI: 190,273] p = 2.2x10^-16^), 1.06 higher odds of experiencing tense feelings[95%CI:1.05,1.07] (p = 3.2x10^-37^) and 0.95 lower odds of playing computer games [95%CI:0.95,0.96] (p = 7.6x10^-30^).

In addition, a 1 SD increased PRS_SCZ_ was associated with 1.05 higher odds of consulting a psychiatrist for nerves, anxiety, tension or depression [95%CI:1.04,1.06] (p = 1.6x10^-17^).

#### Major depressive disorder

PRS_MDD_ was associated with 167 outcomes (Fig 3), including 21 socio-demographic, 84 mental health and 62 general health outcomes.

Most of the associations (74%) were related to mental health, including an association of higher PRS_MDD_ with higher odds of depression, anxiety, irritability, nervousness and mood swings. Strongest evidence of association with PRS_MDD_ was found for 1.08 higher odds of “seen a doctor for nerves, anxiety, tension or depression” [95%CI:1.08,1.09] (p = 2.7x10^-106^), 0.12 points higher neuroticism score [95%CI:0.11,0.14] (p = 2.0x10^-16^) and 1.06 higher odds of having mood swings [95%CI:1.05,1.07] (p = 9.8x10^-58^).

Furthermore, there was strong evidence of 1 SD higher PRS_MDD_ being associated with socio-demographic and lifestyle traits including 1.03 higher odds of ever smoking [95%CI:1.02,1.03] (p = 1.5x10^-13^) and 1.05 higher odds of cannabis use [95%CI:1.03,1.06] (p = 4.1x10^-10^).

Associated physical health measures included 1.03 higher odds of taking medication for pain relief, constipation or heartburn, e.g. paracetamol [95%CI:1.03,1.04] (p = 4.4x10^-11^) and 1.02 odds of more frequent feelings of pain, e.g. back pain [95%CI:1.02,1.03] (p = 1.1x10^-9^).

#### Bipolar disorder

PRS_BP_ was associated with 20 outcomes (Fig 3), including 3 socio-demographic, 14 general health and 3 mental health outcomes.

Socio-demographic and lifestyle factors included associations of higher PRS_BP_ with 1.03 higher odds of having a university degree [95%CI:1.02,1.03] (p = 2.4x10^-12^), 0.02 hours/day less time spent watching television [95%CI:-0.021,-0.010] (p = 2.8x10^-8^) and 0.97 lower odds of playing computer games [95%CI:0.97,0.98] (p = 1.1x10^-9^).

General health traits included 11 traits indicating an association of 1 SD higher PRS_BP_ with 0.06kg/m^2^ lower BMI [95%CI:-0.07,-0.04] (p = 1.6x10^-11^) and 2 traits related to blood measures, such as 0.005% decreased platelet distribution width [95%CI:-0.006,-0.003] (p = 3.1x10^-7^).

Two traits related to mental health were nervous (OR:1.02 [95%CI:1.01,1.03] p = 2.3x10^-7^) and tense feelings (OR:1.02 [95%CI:1.01,1.03] p = 8.0x10^-7^).

### Cross disorder considerations

**Fig 4:**
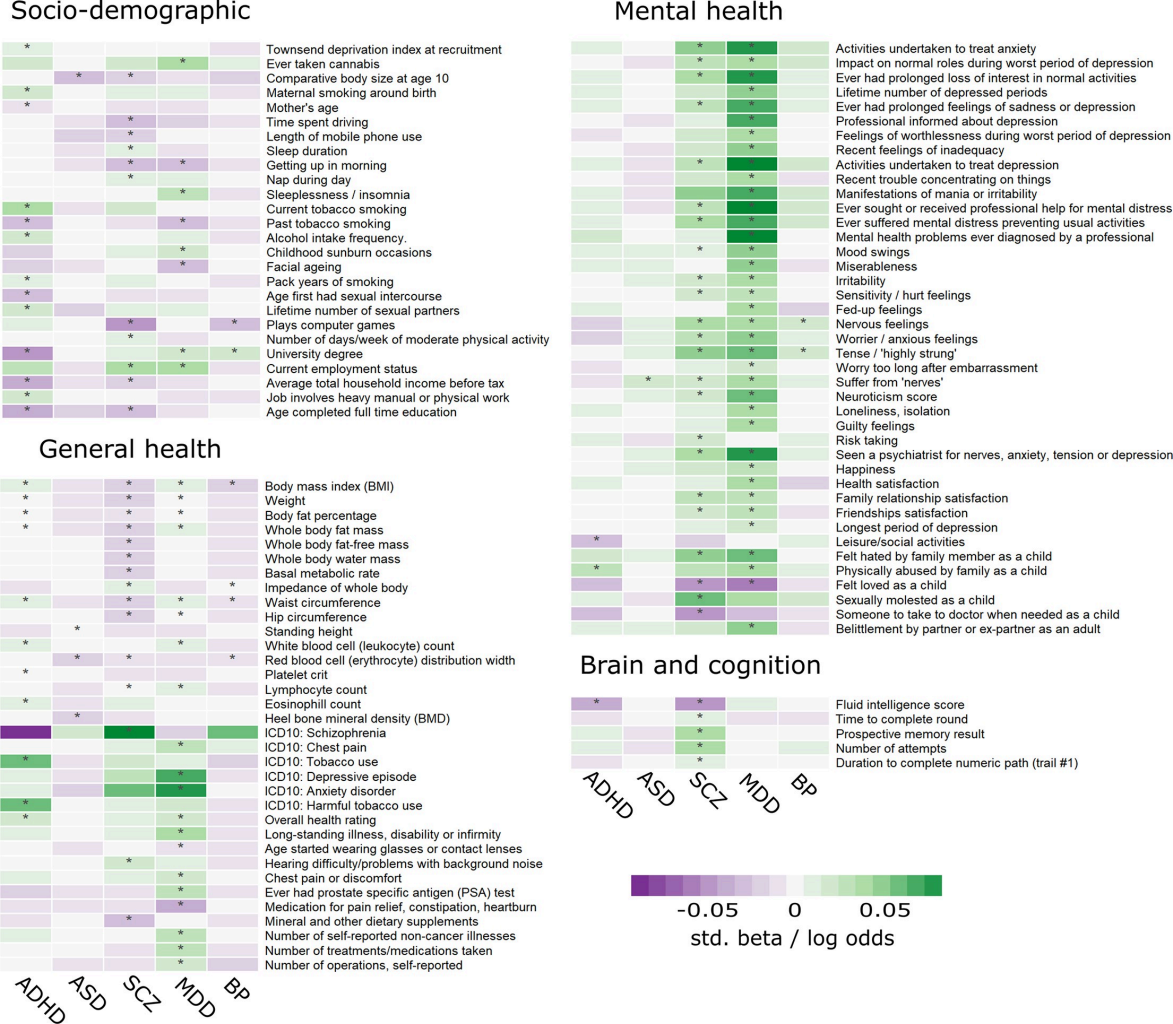
Cross-disorder comparison. Shown are standardized log odds (upper section in each panel) or standardized beta-values (lower section of each panel) of all outcomes associated with polygenic risk scores for either attention deficit/hyperactivity disorder (ADHD), autism spectrum disorder (ASD), schizophrenia (SCZ), major depressive disorder (MDD) or bipolar disorder (BP) at *p*<2.55x10^-6^ as indicated by stars (*). For outcomes categorized as multi-category, only one category is displayed. Only associations with anthropometric measures of the right side of the body are shown. Estimates were generated by PHESANT. Results for continuous outcomes (std. beta) are the standard deviation change of inverse-rank normal transformed outcome per 1 SD higher PRS.

The highest overlap of associated outcomes of the univariable PRS-pheWAS scans was seen between schizophrenia and depression (22 general health, 44 mental health and 9 sociodemographic outcomes in common), bipolar disorder (12 general health, 3 mental health and 2 sociodemographic outcomes in common) and ADHD (18 general health, 1 mental health, 2 brain and cognition and 4 sociodemographic outcomes in common). Large overlap was also seen between ADHD and MDD (22 general health, 3 mental health and 4 socio-economic outcomes) and BP (2 socio-economic and lifestyle and 7 general health outcomes) (Fig 4). However, the majority of the associations are directionally opposite for ADHD and BP. For example, higher PRS_ADHD_ showed evidence for associations with lower educational attainment and higher BMI, whereas higher PRS_BP_ was associated with higher educational attainment and lower BMI.

Furthermore, all disorder PRSs showed some evidence for association with different blood cell counts, such as a decreased leukocyte count for PRS_ADHD_ and PRS_MDD_, or a decreased eosinophil count for PRS_ADHD_ and PRS_SCZ_.

There was very little overlap of highly associated outcomes between the neurodevelopmental domains (ADHD and ASD).

### Sensitivity analysis

We repeated our tests of association for outcomes passing the spectral decomposition threshold, additionally adjusting for potential confounders (assessment centre, genotype chip and the first 40 principal components). These have not been included in the original analysis to reduce the possibility of them introducing collider bias. Estimates were highly consistent with our main results, as shown in S4 and S5 Tables.

Relaxing the p-value threshold for including SNPs in the PRS resulted in some changes in the results (S1 Fig). For ADHD, SCZ, MDD and BP the general trend was an inflation of p-values (S6 Table) and higher effect estimates with smaller confidence intervals. A different pattern was observed for autism spectrum disorder with inconsistent results for some of the outcomes, as described in detail in the supplementary S1 Text. Overall the strength of associations obtained for blood cell count traits across disorders varied between p-value thresholds, with weaker associations found for less stringent p-value thresholds.

When applying the more stringent Bonferroni correction for multiple testing we found that the number of strongly associated outcomes with the PRS reduced slightly to 71 outcomes for PRS_ADHD_, 9 for PRS_ASD_, 155 for PRS_SCZ_, 166 for PRS_MDD_ and 19 for PRS_BP_. Outcomes that did not pass Bonferroni but phenoSPD were “Weight”, “Trunk fat mass” and “Forced expiratory volume in 1 second” for ADHD; “Forced vital capacity (FVC)” for ASD; “Word interpolation” and “Valsartan prescription” for SCZ; “Action taken following self-harm” for MDD and “Spherical power (left)” for BP.

In order to reduce potential biases due to sample overlap with the MDD GWAS from Wray et al. [5], we computed a PRS based on the Wray et al. sample excluding UK Biobank and 23andMe participants (PRS_MDDnoUKB_). The corresponding pheWAS resulted in 51 outcomes passing the multiple testing threshold (S7 Table). Overall, effect estimates were attenuated for many previously identified outcomes and about one third of them did not pass the multiple testing threshold anymore. On the other hand, new associations especially with anthropometric traits could be observed.

## Discussion

In this study, we conducted a PRS-pheWAS to examine the relationships between genetic liability for five major psychiatric disorders and 23,004 outcomes in about 330,000 UK Biobank participants.

**Fig 5:**
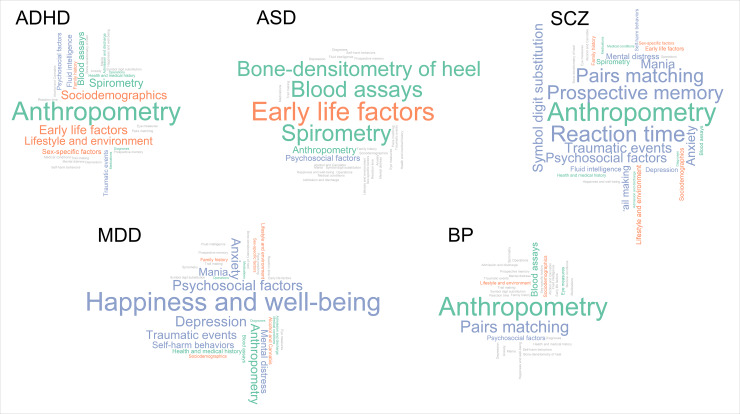
Categories of highly associated outcomes with polygenic risk scores for attention deficit/hyperactivity disorder (ADHD), autism spectrum disorder (ASD), schizophrenia (SCZ), major depressive disorder (MDD) and bipolar disorder (BP). Size of categories depends on the relative number of associated outcomes to the total number of outcomes within each category. Only categories with more than 1 variable are shown. Lifestyle and socio-demographic factors are shown in orange, physical health measures are shown in green and mental health, brain and cognition traits are shown in violet. Grey categories had zero hits for the corresponding disorder.

Our results build on a large body of literature supporting links between genetic risk for psychiatric disorders with a wide variety of outcomes including psychological well-being, lifestyle, socio-demographic factors and physical health [2,4,7,15,16]. Our findings also suggest that although psychiatric disorders show strong genetic overlap [7], genetic risk for distinct psychiatric disorders show differential associations with lifestyle, socio-demographic factors and physical health as highlighted in Fig 5. Genetic liability for ADHD and bipolar disorder showed the strongest associations with lifestyle and social environmental factors as well as physical health. On the other hand, genetic liability for major depression and schizophrenia was most strongly associated with psychological health and associations with lifestyle and socio-demographic factors were less robust.

We were able to replicate previously reported associations between genetic liability for ADHD and lower educational attainment [17,18], higher prevalence of smoking [19], younger age at delivery [20] and higher body mass index [21]. While the previous findings for smoking and BMI were identified in young adults, our findings using an adult population-based sample with a mean age of 56 years, suggest that associations of childhood psychiatric disorder genetic liabilities with health and social outcomes persist into later adulthood. Associations of genetic liability for ADHD in childhood could represent effects of childhood ADHD or sub-threshold ADHD on long-term social and economic outcomes, or alternatively associations could be due to parental effects or horizontal pleiotropy (the same genetic variants affecting multiple traits). Hence, some of the observed associations in this study might also be more likely to act as a risk factor for the corresponding disorder, rather than being the consequence of it.

Interestingly ADHD genetic liability was also associated with a history of physical maltreatment. This result adds to findings from previous twin and adoption designs that have suggested that ADHD and ADHD genetic liability may have “evocative” effects on parent-child hostility [22–24].

Many of the associations of genetic liability for MDD with increased mood swings, irritability, feelings of loneliness and isolation are clinically known and have previously been reported [5]. Our results are also in line with a recent publication from the Brainstorm consortium investigating genetic correlations among psychiatric disorders with neurological and quantitative traits using LD score regression and GWAS summary statistics, reporting high genetic correlations between most psychiatric disorders and educational attainment and BMI [2,7]. However, we found little evidence for associations of genetic liability for ADHD and ASD with mental health outcomes, such as depressive symptoms, neuroticism or anxiety; and very few associations with cognitive or brain imaging outcomes, which might be because of the UK Biobank being a selected sample with lower rates of psychiatric disorders than the general population as discussed in the limitations section.

In addition to identifying previously reported associations, our PRS-pheWAS also revealed novel associations. We found a strong association of genetic liability for ASD with decreased heel bone mineral density, which furthers previous evidence from observational studies that children and adolescents with ASD have lower bone mineral density [25,26], higher frequency of bone fractures [27] and lower vitamin D levels [28,29], which is essential for bone metabolism. This might suggest that these observed associations may be due to pleiotropic effects of genetic variants associated with bone health.

In line with our results, other previous work in schizophrenia patients and their relatives identified an association between schizophrenia and longer performance duration on the Trail Making Test [30], which requires searching and connecting irregularly arranged targets (digits and letters) in ascending order and is widely used to test for executive function, cognitive ability and processing speed [31–36].

Altered blood cell counts were associated with genetic liability for all disorders. Many psychiatric disorders have been previously associated with allergic or inflammatory states [37–39], such as asthma [40] and atopic diseases [41,42] but it is unclear whether high inflammatory states are on the causal pathway to disorder manifestation or the result of comorbid and confounding behaviours associated with the disease, such as restricted diet, overweight, risky behaviours or medication. Our results support the possibility that altered blood cell counts could be a consequence of the disorder, but we cannot rule out contributions of horizontal pleiotropic effects that weaken or intensify the observed association when adding more SNPs into the PRS. Also, considering the inconsistent findings from the sensitivity analyses for blood count traits, results need further validation and should be treated with caution.

In addition to previous observations between psychiatric disorders and later outcomes that we were able to identify in our pheWAS, there are many reported and established associations that we did not observe in our study. For example, we did not observe an association between genetic risk for psychiatric disorders, apart from a negative association with PRS_SCZ_, and type 2 diabetes mellitus (T2D). Reasons for this lack of observations may include selection bias as discussed in the next paragraph, confounding of observational studies or complex relationships between disorders. Although there is evidence of genetic overlap between T2D and SCZ [43] the causal pathway is potentially complex and bidirectional, and needs to be investigated using a formal causal framework. However, being able to identify true causal relationships and shared biological pathways between psychiatric disorders and other health or socio-economic outcomes is essential in designing interventions. Hence, we encourage researchers to triangulate our findings using other causally informative studies.

### Limitations

Patients with psychiatric disorders or high genetic liability for psychiatric disorders are known to be less likely to participate in studies in the first place and more likely to drop-out during an ongoing study [44]. Selection into a study as well as attrition can induce collider bias [45]. There is consensus that the UK Biobank sample is not representative of the UK population, with participants showing, for example, lower prevalence of current smoking and lower rates of mortality [9]. If both having a psychiatric disorder and a specific outcome (e.g. high socio-economic position) are associated with participation (the collider), this can induce an association between genetic risk for psychiatric disorders and the outcome (S2 Fig). Supplementary S8 Table provides an overview of the expected direction of the collider bias on the effect estimates for a simple model with positive or negative association with participation of the exposure or outcome variable, which can be used as a guide to expected direction of bias under some circumstances.

A direct comparison of PHESANT estimates across the psychiatric disorders cannot be done without taking the differentially powered GWASs and derived PRS into account. This can affect the number and set of outcomes associated with each disorder, which only allows for a relative comparison among the PRS. We also cannot calculate the predictive power of the ADHD, ASD, schizophrenia and bipolar PRS in the UK Biobank sample due to very low number of self-reported diagnoses of these disorders. Further, the MDD GWAS used in the current study to calculate genetic risk scores included thirty thousand participants from UK Biobank (about 10% of the GWAS sample) which might have inflated our results for depression related items but is not expected to introduce bias in any other traits, such as blood counts. However, sensitivity analysis using a GWAS excluding the UK Biobank sample and hence much smaller sample size did confirm many of the found observations.

Although genetic risk scores were derived using variants associated at genome-wide significance level, they can still have horizontal pleiotropic effects on different disorders and traits. Hence, our reported associations cannot on their own inform about causality but should be followed up with other causally informative methods to assess the true direction of the causal effect as well as pleiotropy and heterogeneity between genetic variants. We therefore encourage triangulation of results using other study designs [46,47], such as two-sample MR, negative control or twin studies.

### Conclusion

We were able to show that genetic liability for five common psychiatric disorders is associated with distinct domains of adult life, including socio-demographic factors, mental and physical health. This is evident even in individuals from the general population who do not necessarily present with a psychiatric disorder diagnosis, or for individuals who may have been diagnosed as a child but whose symptoms have decreased since. Our research has potential implications for both risk factors and consequences of mental health problems. Our findings indicate potential factors associated with genetic liability for psychiatric disorders, including some which have been identified before (e.g. irritability and depression) and also novel hypotheses (e.g. the association of genetic risk for ASD and reduced bone mineral density) that could be tested using different study designs. Finally the findings also support well-established research into the high long-term economic, societal and individual costs associated with mental health problems [48] and highlight that it is important for mental health scientists and clinicians to consider a broad range of lifestyle, socio-demographic and health risks beyond core diagnoses, in those at elevated familial/genetic risk as well as those with a psychiatric disorder.

## Methods

### Ethics statement

UK Biobank received ethical approval from the research ethics committee (reference 13/NW/0382). All participants provided informed consent to participate. This work was done under application number 16729 (using genetic data version 3 and phenotype dataset 21753).

### Study population

Between 2006–2010 UK Biobank recruited 503,325 men and women in the England, Wales and Scotland at ages 40–69 years. The cohort contains a large dataset including physical measurements, blood/urine/saliva samples, health and lifestyle questionnaires as well as genotype (https://www.ukbiobank.ac.uk/).

**Fig 6:**
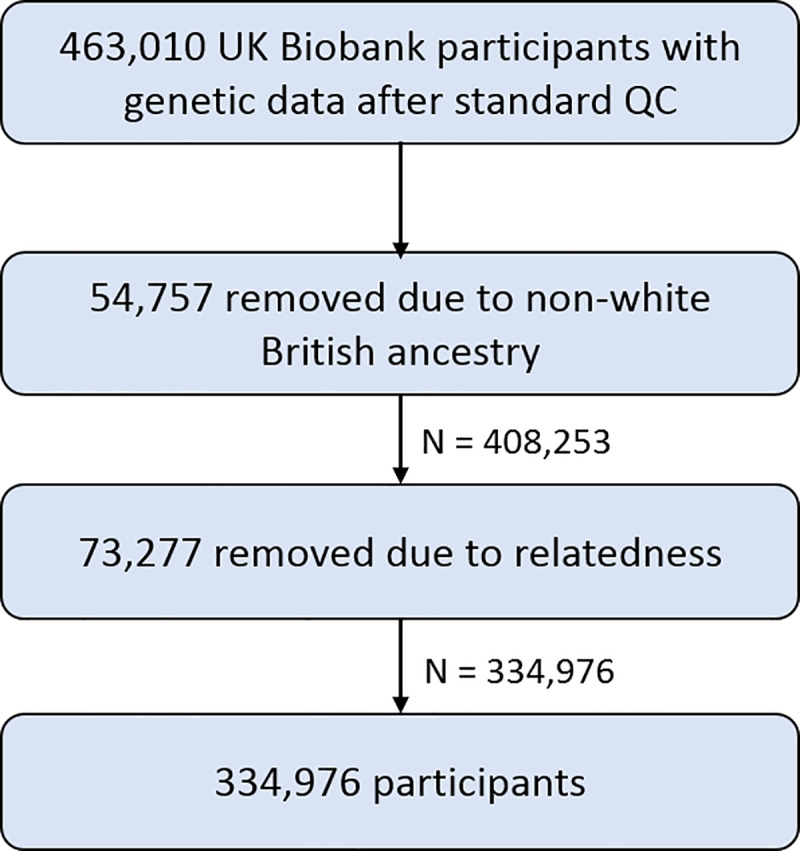
Overview of study sample derivation. Participants with withdrawn consent, sex mismatch or sex aneuploidy were already removed from the dataset in standard QC steps. [49].

For 463,010 participants genotyping was performed using the Affymetrix UK BiLEVE Axiom array or Affymetrix UK Biobank Axiom array. Participants with non-white British ancestry, defined as a self-reported non-white British ancestry in combination with a genetic principal component analysis (PCA) analysis conducted by UK Biobank (n = 54,757 non-white British or non-Caucasian) [49], and participants who had a kinship coefficient denoting a third-degree relatedness (based on Manichaikul et al. [50]; kinship coefficient < 0.0625, n = 73,277) were removed from an already quality checked dataset (excluding participants with withdrawn consent, sex mismatch or sex aneuploidy) [49,50], resulting in a dataset containing 334,976 participants (Fig 6).

### Polygenic risk scores

Genetic variants were identified from the most recent GWAS summary statistics listed in Table 1 with p<5x10^-8^ for ADHD, ASD, SCZ, MDD and BP. This stringent p-value cut-off was chosen to minimize bias introduced by horizontal pleiotropic effects of genetic variants. All summary statistics were subject to standard quality control including filtering for minor allele frequency (MAF>0.1) and imputation quality (INFO>0.8) and excluding the MHC region on chromosome 6 (26-33Mb) due to its complex linkage disequilibrium structure. Polygenic risk scores (PRS) were derived using independent risk alleles at p<5x10^-8^ in approximate linkage disequilibrium (R^2^<0.1 within 500kb distance) and computing a weighted, standardized mean score from these, as has been described previously [51,52].

**Table 1:** Details of GWAS used for calculating PRS.

*disorder*	*cases*	*controls*	*SNPs in PRS*^*1*^	*Heritability*	*Source*
ADHD	20,183	35,191	10	5.5%^2^	Demontis et al. (2019) [2]
ASD	18,381	27,969	2	2.5%^2^	Grove et al. (2017) [4]
Schizophrenia	36,989	113,075	99	7%^3^	Ripke et al. (2014) [3]
MDD	135,458	344,901	44	1.9%^3^	Wray et al. (2018) [5]
Bipolar disorder	20,129	21,524	8	4%^3^	Ruderfer et al. (2018) [53]

1- PRS derived from genome-wide significant hits (p<5x10^-8^)

2- SNP heritability estimates (Nagelkerke’s R2) reported in the corresponding discovery sample

3-Percent of variance on the liability scale explained by PRS reported in corresponding discovery sample

ADHD–Attention deficit/hyperactivity disorder, MDD–Major depression, ASD–Autism spectrum disorder

### Outcomes

UK Biobank provides a fully searchable data showcase (http://biobank.ctsu.ox.ac.uk/crystal/) which at the time of data download (March 2018) included 23,004 outcomes (see supplementary S2 Text), including lifestyle and environment, socio-demographic, early life factors, anthropometry, family history and depression outcomes.

Age, sex and the first 10 principal components derived from the genetic data were included as covariates in all regression models. Age was derived from the participants date of birth and the date of their first assessment centre visit. Sex was self-reported and validated using genetic data.

### PHESANT PRS-pheWAS

PHESANT package (version 0.17) was used to test the association of each PRS with each outcome variable in Biobank. A detailed description of PHESANT’s automated rule-based method is given elsewhere [54,55]. In brief, decision rules are based on the variable field type and categorize each variable as one of four data types: continuous, ordered categorical, unordered categorical or binary. PHESANT then estimates the univariate association of the PRS (independent) with each outcome variable (dependent) in a regression model, respectively. Normality of continuous data is ensured by an inverse normal rank transformation prior to testing. To compute meaningful and better interpretable estimates, outcomes passing the multiple testing threshold that qualified as continuous outcomes were followed up on their original scale using a linear regression, excluding outliers and checking for normal distribution of residuals. All estimates correspond to 1 SD change of the PRS.

PHESANT assigns each UK Biobank outcome to one of 91 level 3 categories based on the 235 origin categories of the UK Biobank catalogue (a full list of categories is provided in S1 Table). Furthermore, three authors (BL, EW, ES) grouped these 91 categories into four prespecified higher level categories in order to aid result presentation: socio-demographics and lifestyle, brain and cognition, mental health and general health (Fig 1B).

To account for multiple testing (n = 23,004 tests) we used a previously derived threshold [55,56] based on an estimate of the number of independent phenotypes calculated using spectral decomposition (phenoSPD) (n = 19,645). The multiple testing adjusted significance threshold was p<2.55x10^-6^ (0.05/19,645). The amount of inflation of observed versus expected p-values is given as the ratio of the median chi-squared statistics for observed to expected median p-values, referred to as Lambda (λ). A conservative Bonferroni correction of multiple testing that assumes uncorrelated traits, would yield a similar p-value threshold of p<2.17x10^-6^ (0.05/23,004).

### PHESANT sensitivity analysis

Analyses were re-run to assess residual confounding of assessment centre and genetic batch, including them as well as all 40 principal components as additional covariates for outcomes identified as strongly associated with either one of the disorders PRS. These covariates were not included in the first model because this could introduce collider bias if, for example, location of assessment centre is affected by both genetic predisposition and outcomes, as discussed in the limitations section.

Furthermore, PRS were derived using various p-value thresholds (p<0.01, p <0.1x10^-3^, p<1x10^-4^, p<1x10^-5^, p<1x10^-6^) with consequently increasing numbers of SNPs (S9 and S10 Tables) and the five PRS-pheWAS were re-run with the more relaxed PRS to capture a larger amount of explained variation in the disorders by accepting an increase in horizontal pleiotropic effects and adjunct noise. For MDD GWAS results were available for only 10,000 SNPs at these additional thresholds due to availability restrictions.

To assess biases due to a sample overlap between the Wray et al. MDD GWAS and UK Biobank, we computed a PRS based on the Wray et al. sample excluding the UK Biobank and 23andMe samples (43,204 cases and 95,680 controls, n_SNPs_ in PRS = 3) as described in Howards et al. [57] and re-run the pheWAS based on this.

All analyses were performed in R version 3.2.4 ATLAS and R version 3.3.1, and the code is available at [https://github.com/MRCIEU/Psychiatric-disorder-pheWAS-UKBB]. Git tag v0.2 corresponds to the version presented here.

## Supporting information

S1 Fig Top associated outcomes with PRS for attention deficit/hyperactivity disorder (ADHD), autism spectrum disorder (ASD), schizophrenia (SCZ), bipolar disorder (BP) and major depressive disorder (MDD) across different p-value thresholds for SNP inclusion (5x10^-8^ to 1x10^-2^). (TIFF)

S2 Fig Schematic description of collider bias and confounding when testing for associations between genetic liability for psychiatric disorders and adulthood outcomes in UK Biobank. (TIFF)

S3 Fig Histograms of standardized PRS for attention deficit/ hyperactivity disorder (ADHD, n_SNP_ = 10), autism spectrum disorder (ASD, n_SNP_ = 2), schizophrenia (SCZ, n_SNP_ = 113), major depression (MDD, n_SNP_ = 39) and bipolar disorder (BP, n_SNP_ = 8). (TIFF)

S1 Table Overview of UK Biobank categories with total number of outcomes per category and number of outcomes associated with polygenic risk scores (with p-value below significance threshold of 2.55x^10-6^). (ADHD- attention defict/ hyperactivity disorder, ASD- autism spectrum disorder, SCZ- schizophrenia, MDD- major depressive disorder, BP- bipolar disorder)
(XLSX)

S2 Table Pearson correlation matrix of polygenic risk scores (p<5x10^-8^). Correlation coefficients are displayed on the left side, p-values on the right side of the table. (ADHD- attention defict/ hyperactivity disorder, ASD- autism spectrum disorder, SCZ- schizophrenia, MDD- major depressive disorder, BP- bipolar disorder)
(XLSX)

S3 Table PRS-pheWAS results for association of genetic risk of 5 common psychiatric disorders with 23,004 outcomes in UK Biobank. Genetic risk scores were calculated as the weighted sum of all genome-wide significant risk alleles for each disorder. Estimates were generated by PHESANT. Results for continuous outcomes are the standard deviation change of inverse-rank normal transformed outcome per 1 SD higher PRS.
(XLSX)

S4 Table PRS-pheWAS follow-up and sensitivity results for selected continuous outcomes. Genetic risk scores were calculated as the weighted sum of all genome-wide significant risk alleles for each disorder. Estimates were generated by linear regression on the original variable scale per 1 SD higher PRS.
(XLSX)

S5 Table PRS-pheWAS sensitivity results for association of generic risk of 5 common psychiatric disorders with 23,004 outcomes in UK Biobank, additionally adjusted for genetic batch and assessment centre. Genetic risk scores were calculated as the weighted sum of all genome-wide significant risk alleles for each disorder.
(XLSX)

S6 Table Number of strongly associated traits with PRS for attention-deficit/hyperactivity disorder (ADHD), autism spectrum disorder (ASD), schizophrenina (SCZ), major depressive disorder (MDD) and bipolar disorder (BP) at different p-value thresholds for PRS calculation. (XLSX)

S7 Table PRS-PheWAS results for association of genetic risk of major depression with 23,004 outcomes in UK Biobank. Genetic risk score was calculated as the weighted sum of all genome-wide significant risk alleles for major depression using full GWAS summary statistics as reported in Wray et al. 2019 and GWAS summary statistics excluding the UK Biobank and 23andMe sample from the mentioned study (noUKB). Estimates were generated by PHESANT. Results for continuous outcomes are the standard deviation change of inverse-rank normal transformed outcome per 1SD higher PRS.
(XLSX)

S8 Table Expected bias of effect estimates due to association of exposure and outcome variable with participation in a study (collider) under simplified assumptions. (XLSX)

S9 Table Number of SNPs included in polygenic risk scores for attention-deficit/hyperactivity disorder (ADHD), autism spectrum disorder (ASD), schizophrenina (SCZ), major depressive disorder (MDD) and bipolar disorder (BP) at different p-value thresholds. (XLSX)

S10 Table Correlation (Pearson's correlation coefficient) of polygenic risk scores computed at different p-value thresholds. (XLSX)

S1 Text Sensitivity analysis for autism spectrum disorder. (DOCX)

S2 Text UK Biobank outcomes description. (DOCX)
