# Supplementary figures and images for: A cross-disorder PRS-pheWAS of 5 major psychiatric disorders in UK Biobank
Source: PLoS Genet. 2020 May 11;16(5):e1008185. doi: 10.1371/journal.pgen.1008185 (PMC7274459; doi:10.1371/journal.pgen.1008185)

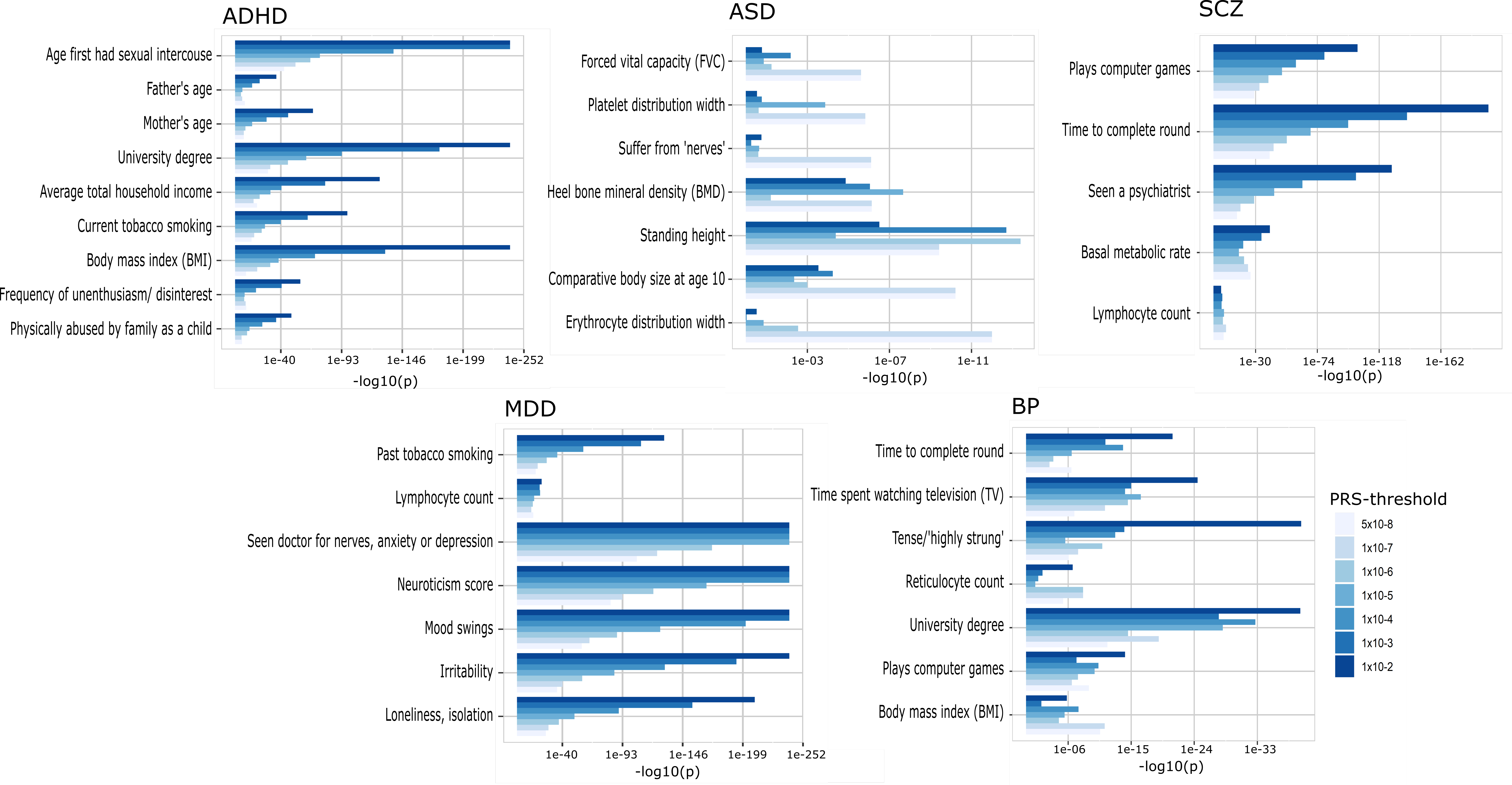

Supplement: S1 Fig — (TIFF) [file pgen.1008185.s001.tiff]

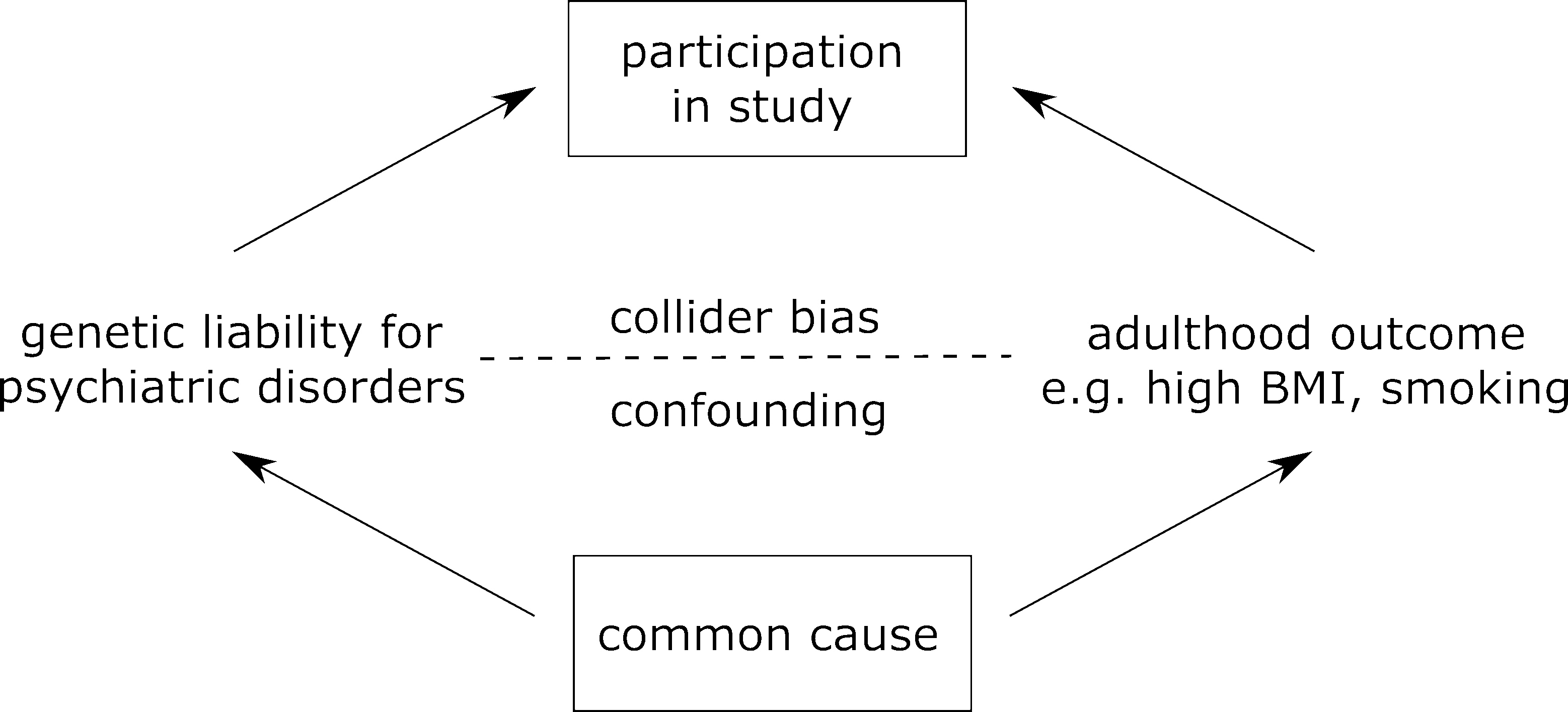

Supplement: S2 Fig — (TIFF) [file pgen.1008185.s002.tiff]

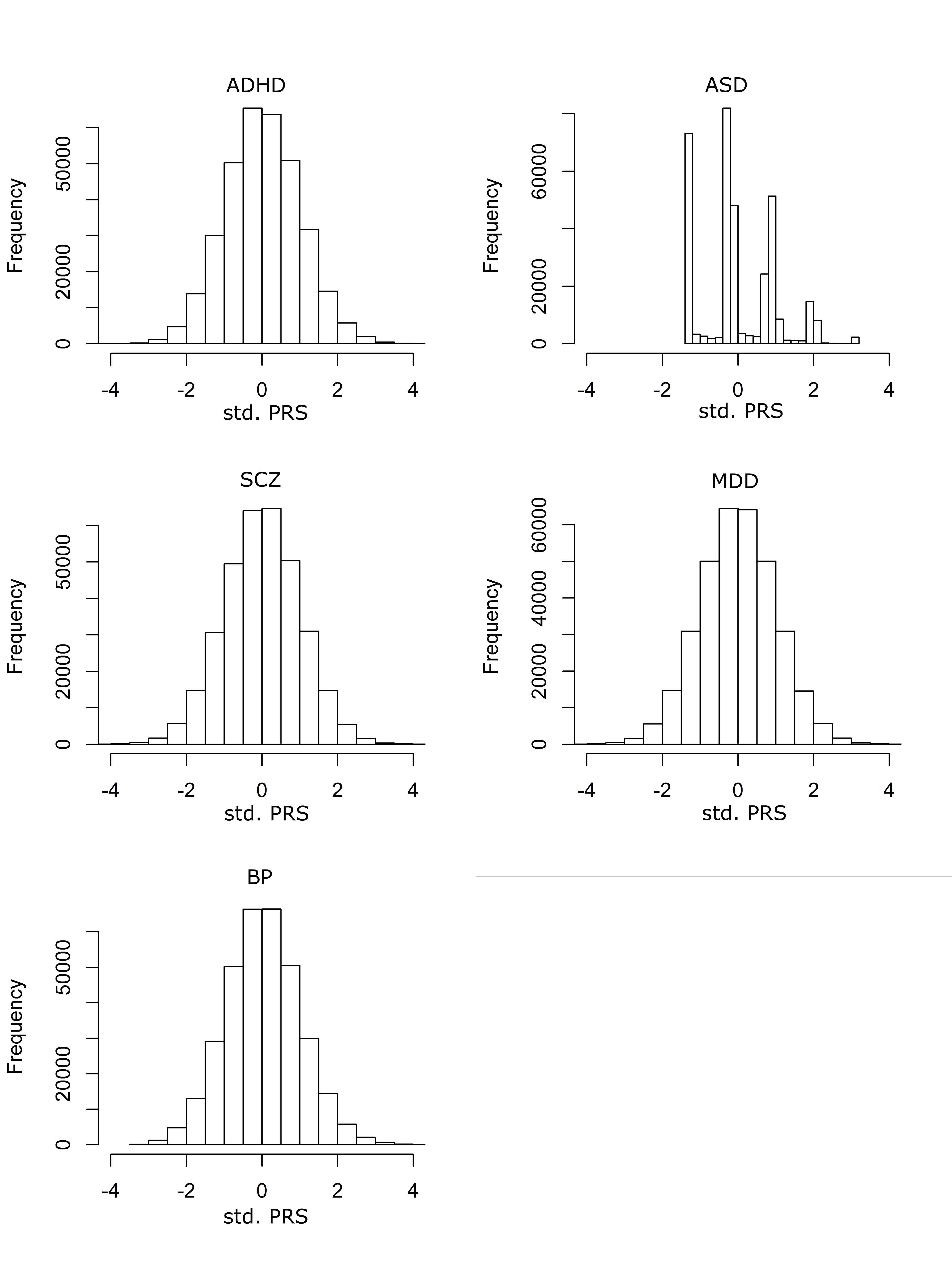

Supplement: S3 Fig — (TIFF) [file pgen.1008185.s003.tiff]
