# Supplementary material for: A cross-disorder PRS-pheWAS of 5 major psychiatric disorders in UK Biobank
Source: PLoS Genet. 2020 May 11;16(5):e1008185. doi: 10.1371/journal.pgen.1008185 (PMC7274459; doi:10.1371/journal.pgen.1008185)
Supplement: S1 Text — (DOCX) [file pgen.1008185.s014.docx]

Supplementary material

**A cross-disorder PRS-pheWAS of 5 major psychiatric disorders in UK Biobank**

Beate Leppert, Louise AC Millard, Lucy Riglin, George Davey Smith, Anita Thapar, Kate Tilling, Esther Walton, Evie Stergiakouli

**Text S1. Sensitivity analysis for autism spectrum disorder**

Lowering the *p*-value threshold for sensitivity analysis for autism spectrum disorder resulted in inconsistent associations for some of the outcome. About half of the associated traits showed an inflation of p-values for PRS thresholds of p<1x10^-4^ and higher. For example, PRS_ASD_ showed strong evidence for an association with “Having a university degree” at p<1x10^-2^–1x10^-4^ (p=3.0x10^-8^, 1.5x10^-17^, 6.3x10^-29^, respectively) but not at 1x10^-5^–5x10^-8^ (p=8.4x10^-3^,1.4x10^-3^, 6.1x10^-1^, 6.1x10^-1^, respectively). However, other traits showed inconsistent results such as for bone mineral density, which showed high evidence for association with PRS_ASD_ at p<5x10^-8^, p<1x10^-7^, p<1x10^-5^ and p<1x10^-4^ (p=7.3x10^-7^, 7.3x10^-7^, 2.2x10^-8^,8.8x10^-7^, respectively) but not at p<1x10^-6^, p<1x10^-3^ and p<1x10^-2^ (p=6.0x10^-2^, 1.4x10^-5^, 5.2x10^-4^, respectively).
