# Supplementary material for: A cross-disorder PRS-pheWAS of 5 major psychiatric disorders in UK Biobank
Source: PLoS Genet. 2020 May 11;16(5):e1008185. doi: 10.1371/journal.pgen.1008185 (PMC7274459; doi:10.1371/journal.pgen.1008185)
Supplement: S2 Text — (DOCX) [file pgen.1008185.s015.docx]

Supplementary material

**A cross-disorder PRS-pheWAS of 5 major psychiatric disorders in UK Biobank**

Beate Leppert, Louise AC Millard, Lucy Riglin, George Davey Smith, Anita Thapar, Kate Tilling, Esther Walton, Evie Stergiakouli

**Text S2. UK Biobank outcomes description**

UK Biobank provides a fully searchable data showcase to identify variables based on the field type (integer, continuous, categorical (single) and categorical (multiple)). There were 2,143 fields at the time of data download (March 2018). We removed fields identifying the assessment centre environment (18), fields described as ‘polymorphic’ (2), 13 genetic descriptor fields, 1 sex field and 4 age fields were removed. Furthermore, 18 categorical (single) fields with more than one value per person were removed and 14 fields were not currently available, which results in a total of 23,004 outcomes.
